# Stanford type a aortic dissection with cerebral infarction: a rare case report

**DOI:** 10.1186/s12883-020-01832-y

**Published:** 2020-06-23

**Authors:** Jie Wang, Li-Rong Wu, Xin Xie

**Affiliations:** Department of Neurology, Chongqing City Hospital of Traditional Chinese Medicine, No. 6, Seventh Branch Road, Panxi, Jiangbei District, Chong qing, 400021 China

**Keywords:** Aortic dissection, Cerebral infarction, Intravenous thrombolysis

## Abstract

**Background:**

Aortic dissection (AoD) is a disease with a high mortality rate. Its clinical manifestations are diverse and covert, which makes diagnosis and treatment challenging. Here, we report a very rare case of aortic dissection leading to bilateral cerebral cortex ischaemia and epilepsy.

**Case presentation:**

A 54-year-old man was admitted to the hospital with acute onset of right limb weakness accompanied by slurred speech. He had a history of hypertension as well as tobacco and alcohol use. The patient was found to have aphasia and right hemiplegia on physical examination. No bleeding was seen on the skull CT. Acute cerebral infarction was considered after admission, and rt-PA was administered for intravenous thrombolysis. During intravenous thrombolysis, the patient suddenly developed epilepsy, and diazepam was given immediately by intravenous injection to control the symptoms. Emergency skull diffusion-weighted imaging (DWI) was performed, and the results showed a small, patchy, high signal that was scattered throughout the left brain hemisphere, right frontal parietal lobe and centrum semiovale. Head and neck CT angiography (CTA) was performed; dissection was found in the ascending aorta, aortic arch, bilateral common carotid artery, proximal part of the internal carotid artery, and initial segment of the left external carotid artery. The laceration was located in the upper part of the ascending aorta. AoD complicated by acute cerebral infarction and epilepsy was considered, and the patient was immediately transferred to the cardiovascular surgery specialist hospital for surgical treatment.

**Conclusions:**

Some aortic dissections have no typical manifestations of chest pain, and the onset is covert. Atypical clinical manifestations of epilepsy secondary to bilateral cerebral hemisphere infarction may appear. AoD with cerebral infarction is a contraindication for intravenous thrombolysis; surgical treatment is the best way to reduce mortality.

## Background

Aortic dissection (AoD) is a disease with a high mortality rate and an incidence of 2.5–3.5/100,000 people per year [1, 2]. Without early surgical intervention, the mortality of Stanford type A AoD at 3 days after onset is greater than 50% [3]. The most common initial manifestation of AoD is pain; however, there are certain patients with AoD who mainly present with neurological symptoms but do not have pain. These patients are likely to be missed clinically.

The latest guidelines currently treat acute cerebral infarction with AoD as a contraindication to intravenous thrombolytic therapy [4]. If AoD is not ruled out before intravenous thrombolytic therapy, the consequences will be disastrous and may result in a high mortality rate and poor prognosis [5]. A case of AoD with unusual cerebral infarction occurring at our hospital has now been analysed and reported; it is hoped that this case will serve as a reference for clinical practice.

## Case presentation

The patient was a 54-year-old middle-aged man who experienced acute-onset symptoms. He was admitted to the hospital due to acute onset of weakness in his right limbs accompanied by slurred speech for 2 h. He had a history of hypertension as well as tobacco and alcohol use. The admission examination revealed consciousness, incomplete mixed aphasia, and grade 4 right upper and lower limb muscle strength. No bleeding was seen on the skull CT. Blood tests showed D-dimer 177.60 mg/l and C-reactive protein 130.8 mg/l, and routine blood tests showed white blood cells 11.43 × 10^9^/l, red blood cells 3.2 × 10^12^/l, and haemoglobin 10^6^ g/l.

**Fig. 1:**
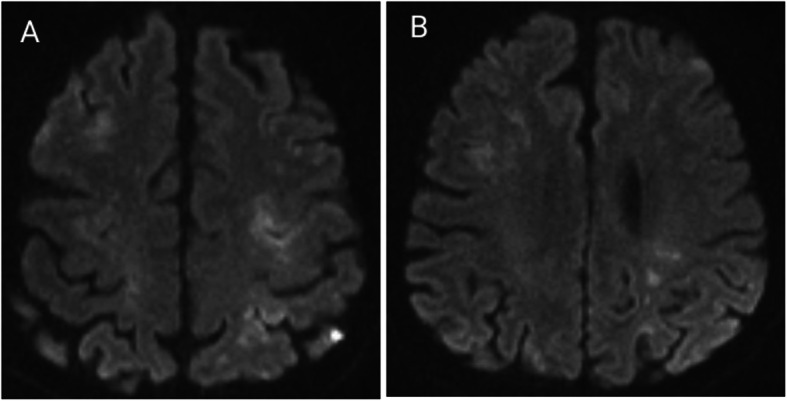
DWI of the patient showed small patchy high signals scattered throughout the left brain hemisphere and right frontal parietal lobe

**Fig. 2:**
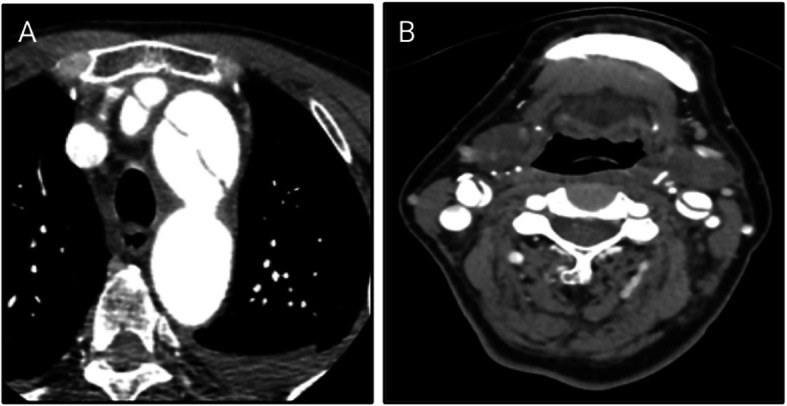
CT angiography (CTA) images of the patient showed dissection of the ascending aorta, aortic arch and bilateral common carotid arteries

Acute cerebral infarction was considered after admission. Recombinant tissue plasminogen activator (rt-PA; 45 mg) was administered for intravenous thrombolysis. During intravenous thrombolysis, the patient suddenly lost consciousness and exhibited involuntary convulsions in the limbs accompanied by increased salivation. Intravenous thrombolysis was immediately stopped, and 10 mg diazepam was given by intravenous injection. Emergency CT scan of the skull showed no bleeding after the cessation of convulsions. Emergency skull diffusion-weighted imaging (DWI) was performed 1 h after intravenous thrombolysis, and the results showed a small patchy high signal that was scattered throughout the left brain hemisphere, right frontal parietal lobe and centrum semiovale (Fig. 1). Therefore, acute cerebral infarction was considered. Head and neck CT angiography (CTA) was performed, and dissection was found in the ascending aorta, aortic arch, bilateral common carotid artery, proximal part of the internal carotid artery, and initial segment of the left external carotid artery. The laceration was located in the upper part of the ascending aorta (Fig. 2). Stanford type A AoD complicated by acute cerebral infarction and epilepsy was considered, and the patient was immediately transferred to the cardiovascular surgery specialist hospital for surgical treatment.

## Discussion and conclusions

The pathogenesis of AoD is currently unclear, and the most common causes of AoD are hypertension and atherosclerosis, which account for approximately 80% of dissection cases. Other factors include trauma, Marfan syndrome, iatrogenic factors, bicuspid aortic valve deformities, familial asymptomatic aortic dissection, intramural haematoma expansion, pregnancy and arteritis [6]. This patient had no obvious history of trauma, but there was a history of untreated hypertension. Therefore, the possible causes of dissection were hypertension and atherosclerosis.

The primary manifestation of AoD is often sudden and persistent chest and back pain that cannot be relieved [7]. Approximately 1/3–1/2 of aortic dissections with neurological symptoms have no pain symptoms [8, 9]. The possible reason is that these patients often exhibit a disturbance of consciousness, aphasia and/or amnesia, and their ability to communicate is lost [5]. Clinical symptoms are closely related to dissection initiation and pathophysiological development. This patient presented with a bilateral common carotid artery dissection and first showed right hemiplegia. It is speculated that emboli in left common carotid artery dissections are unstable and easily dislodged, while right common carotid artery dissections are asymptomatic. Therefore, signs of damage to the left brain hemisphere were first manifested clinically in our patient. When alteplase was used for intravenous thrombolysis, the bilateral common carotid artery intimal thrombus disintegrated and dislodged, resulting in extensive embolization events in both hemispheres and eventually, secondary epilepsy.

Some auxiliary examinations can also provide some clinical information. For example, 80% of patients with aortic dissection have a widened mediastinum and abnormal aortic shape when tested with chest X-ray [8]; echocardiography can prompt arterial lumen intimal stripping. Laboratory D-dimer tests can also provide valuable diagnostic information. A study showed that D-dimer levels were significantly higher in patients with ischemic stroke and AoD than in patients without AoD. This trend was evident within 6 h of the onset of symptoms [10]. Weber et al. found that elevated D-dimer levels are related to the severity of AoD [11].

Currently, the guidelines have recommended intravenous thrombolysis as the highest level of therapy for cerebral infarction within the time window [4]. At this stage, there are many hospitals in China that can perform intravenous thrombolysis, but the level of diagnosis and treatment is uneven. If a patient has AoD, intravenous thrombolysis can have catastrophic consequences, such as new embolic events caused by the disintegration of emboli in the dissection, enlargement of haematomas in the wall of the dissection or around the aorta, pericardial effusion and tamponade due to aortic rupture, and the delay of life-saving surgery [12]. Some AoD patients with neurological defects as the first symptoms have no pain (or the pain cannot be described). Such patients are prone to a missed or delayed diagnosis of AoD. Doctors should also pay attention to any unexplained hypotension, mild dyspnoea, chest discomfort, asymmetry of blood pressure between arms (more than 20 mmHg difference in systolic blood pressure), loss of consciousness, changes in the electrocardiogram, cardiac murmurs, and cold limbs [13]. If the above symptoms are present, aortic CT angiography and echocardiography should be supplemented to determine whether AoD is present.

Surgery is still the best treatment to reduce the mortality of Stanford type A AoD [14]. Whether neurological complications will affect the prognosis of patients with AoD is still controversial. AoD patients need to maintain a low blood pressure to prevent further tearing or even rupture of the dissection, while low blood pressure will reduce stroke hemisphere perfusion and then enlarge the cerebral infarction [15]. Some researchers believe that concurrent ischaemic stroke is a predictor of poor prognosis in patients with AoD [16, 17]; however, with early diagnosis of AoD and appropriate surgical treatment, concurrent neurological symptoms have been reported to not be associated with increased mortality [8, 18, 19].

Stanford type A AoD is a high-risk fatal disease. The key to controlling it lies in early detection, early diagnosis, and timely surgical treatment. Approximately 1% of patients with ultra-acute ischaemic stroke have aortic dissection [20]. Prior to intravenous thrombolysis, it is necessary to increase risk awareness and exclude AoD as much as possible to avoid iatrogenic damage to patients.

## Data Availability

All data related to this case report are documented within this manuscript.

## Abbreviations

AoD: Aortic dissection
DWI: Diffusion weighted imaging
CTA: CT angiography
rt-PA: Recombinant tissue plasminogen activator
